# Supplementary material for: An Imine-Based Porous 3D Covalent Organic Polymer as a New Sorbent for the Solid-Phase Extraction of Amphenicols from Water Sample
Source: Molecules. 2023 Apr 7;28(8):3301. doi: 10.3390/molecules28083301 (PMC10145516; doi:10.3390/molecules28083301)
Supplement: Supplementary file 1 [file molecules-28-03301-s001.zip › molecules-2291893-supplementary.pdf]

Supplementary Materials

An Imine-based Porous 3D Covalent Organic Polymer as a New Sorbent for the Solid-phase Extraction of Amphenicols from Water Samples

Jinjian Wei[1,*,#], Lengbing Chen[1,#], Rui Zhang[1], Yi Yu[1], Wenhua Ji[2], Zhaosheng Hou[1], Yuqin Chen[1,*], Zhide Zhang[1,*]

[1]College of Chemistry, Chemical Engineering and Materials Science, Key Laboratory of Molecular and Nano Probes, Ministry of Education, Collaborative Innovation Center of Functionalized Probes for Chemical Imaging in Universities of Shandong, Shandong Normal University, Jinan 250014, PR China

[2]Key Laboratory for Applied Technology of Sophisticated Analytical Instruments of Shandong Province, Shandong Analysis and Test Center, Qilu University of Technology (Shandong Academy of Sciences), Jinan, 250014, China

*Correspondence: jinjian.wei@sdnu.edu.cn (J.W.); chenyuqin@sdnu.edu.cn (Y.C.); zdzhang@sdnu.edu.cn (Z.Z.); Tel.: +86-0531-86186003 (Z.Z.)

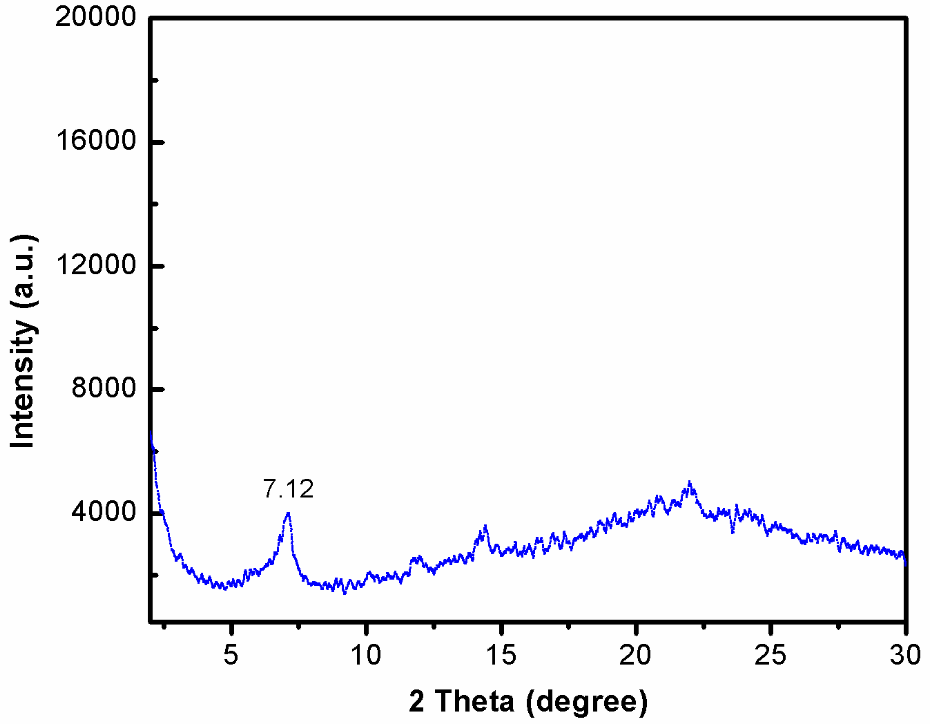

Figure S1. PXRD for imine-based 3D COP

Table S1. RSD of analytical data for determination of amphenicols in water samples.

Added (ng/mL)	RSD（n=3, %）		
	CAP	FF	TAP
0.5	2.49	1.59	3.73
10	3.77	3.14	6.22
100	7.02	2.86	5.34
150	4.16	4.11	3.00
